# Structure-Based Screening of Antiviral Candidates Against Infectious Spleen and Kidney Necrosis Virus (ISKNV) Using Molecular Docking and *In Vitro* Evaluation

**DOI:** 10.3390/biom16071022

**Published:** 2026-07-13

**Authors:** Eun-Jin Baek, Hyun-Deok Choi, Ye-Jin Jeong, Kwang-Il Kim

**Affiliations:** 1 Department of Aquatic Life Medicine, Pukyong National University, Busan 48513, Republic of Korea; eungene8803@gmail.com (E.-J.B.); 201513346@pukyong.ac.kr (Y.-J.J.); 2 Pathology Research Division, National Institute of Fisheries Science, Busan 46083, Republic of Korea; chi943371@naver.com

**Keywords:** infectious spleen and kidney necrosis virus, antiviral activity, structure-based screening, molecular docking, *in vitro* evaluation

## Abstract

Infectious spleen and kidney necrosis virus (ISKNV) causes systemic infections and high mortality in various freshwater and marine fish species, posing a serious threat to aquaculture and ornamental fish industries. In this study, 72 antiviral compounds were screened against five ISKNV target proteins, DNA polymerase, transcription elongation factor (TFIIS), adenosine triphosphatase (ATPase), ankyrin repeat-containing protein, and major capsid protein (MCP) using molecular docking, followed by *in vitro* evaluation in dwarf gourami fin (DGF) cells. Fludarabine, liquiritin, and lycorine consistently ranked among the top 20 compounds across all five targets and were selected for further evaluation. Fludarabine and lycorine strongly inhibited MCP, ATPase, and DNA polymerase expression with inhibition rates exceeding 85%. Infectious viral titers were reduced by approximately 39- and 81-fold in the fludarabine- and lycorine-treated groups, respectively, compared with those in the ISKNV-infected control group. Time-course analysis revealed that both compounds suppressed extracellular viral release and delayed the progression of cytopathic effects. These results suggest that multi-target docking combined with *in vitro* evaluation may be a useful strategy for prioritizing antiviral candidates against ISKNV.

## 1. Introduction

The infectious spleen and kidney necrosis virus (ISKNV) is a double-stranded DNA virus belonging to the genus *Megalocytivirus* within the family *Iridoviridae* [1,2]. ISKNV is known to infect a wide range of freshwater and marine fish species, causing systemic infection, splenomegaly, abnormal swimming behavior, severe tissue necrosis, and high cumulative mortality [3,4,5]. Since its first identification in Mandarin fish in China, ISKNV has been detected in various fish species in several Asian countries, including China, Singapore, Taiwan, Malaysia, and Indonesia [4,6]. More recent reports have described ISKNV infections in India, Australia, Germany, the USA, and Ghana, indicating an expanding geographical distribution [4,7,8,9,10]. ISKNV has also been detected in imported ornamental fish, suggesting that the international ornamental fish trade may contribute to the transboundary dissemination of the virus. In Korea, ISKNV infection was previously reported in freshwater ornamental fish imported from Asian countries, and the virus was recently redetected and isolated from a dwarf gourami (*Trichogaster lalius*) imported from Singapore in 2023 [5,11]. These findings highlight the need for effective disease prevention and antiviral intervention strategies against ISKNV.

To control Megalocytivirus-associated diseases, several vaccination strategies, including formalin-inactivated, recombinant, and DNA vaccines, have been investigated, particularly against the red sea bream iridovirus (RSIV) and ISKNV [12,13,14]. Formalin-inactivated vaccines against RSIV have been developed and applied in several cultured marine fish species, contributing to disease reduction in aquaculture [15,16]. However, vaccine-based control remains primarily preventive and may be limited by the host species, administration method, outbreak timing, and variable protective efficacy among different viral genotypes [2,12]. In addition, antiviral agents have not been commercially established for ISKNV infection, despite the need for rapid intervention during outbreaks. Therefore, identifying effective antiviral candidates against ISKNV remains an important objective in fish virology and aquaculture disease management.

Various approaches have been explored for discovering antiviral compounds, including high-throughput screening, natural compound screening, drug repurposing, and structure-based virtual screening [17,18,19]. Among these, molecular docking has gained prominence as a rapid and cost-effective tool for predicting ligand–protein interactions and prioritizing potential antiviral candidates before biological validation [19,20,21]. Previous studies have also demonstrated that predictive modeling approaches, including molecular docking, can provide an efficient framework for prioritizing therapeutic candidates across diverse disease models and viral pathogens [20,21]. The large ISKNV genome, which contains more than 120 predicted open reading frames (ORFs), necessitates rational target selection in structure-based antiviral screening [1]. Because many ISKNV proteins have not yet been functionally characterized, prioritizing the viral proteins involved in replication, transcription, virion formation, and host interactions may improve the efficiency and biological relevance of docking-based antiviral discovery. Docking-based antiviral screening has been applied to multiple viral pathogens, including the influenza virus, herpesviruses, coronaviruses, and dengue virus [21,22,23,24]. Recent studies applied docking-based methods to viruses affecting aquatic animals, highlighting the potential value of this strategy in fish antiviral research [25]. However, docking predictions alone are insufficient to fully explain antiviral effectiveness because computational interaction scores do not always correlate with actual viral inhibition under biological conditions [26,27].

Because the experimental evaluation of large compound libraries is time-consuming and labor-intensive, structure-based molecular docking provides an efficient strategy for prioritizing potential antiviral candidates for subsequent biological validation. Therefore, confirming the antiviral potential of docking-selected candidates requires biological validation using suitable infection models. In this study, 72 antiviral compounds were screened against five ISKNV target proteins using a multi-target molecular docking approach. To evaluate the biological relevance of the docking-based selection, the selected compounds were further evaluated using an *in vitro* dwarf gourami fin (DGF) cell infection model through cytotoxicity analysis to assess compound safety, viral gene expression assays to determine transcriptional inhibition, infectious viral titer quantification to evaluate productive virus replication, time-course cytopathic effect (CPE) observation to monitor virus-induced cellular damage, and extracellular viral load analysis to assess viral release. Together, these complementary assays provided a comprehensive biological evaluation of the docking-selected candidate compounds. This study aimed to evaluate the applicability of docking-based antiviral screening for ISKNV and identify potential antiviral candidates for further investigation.

## 2. Materials and Methods

### 2.1. Platform for Molecular Modeling

**Figure 1:**
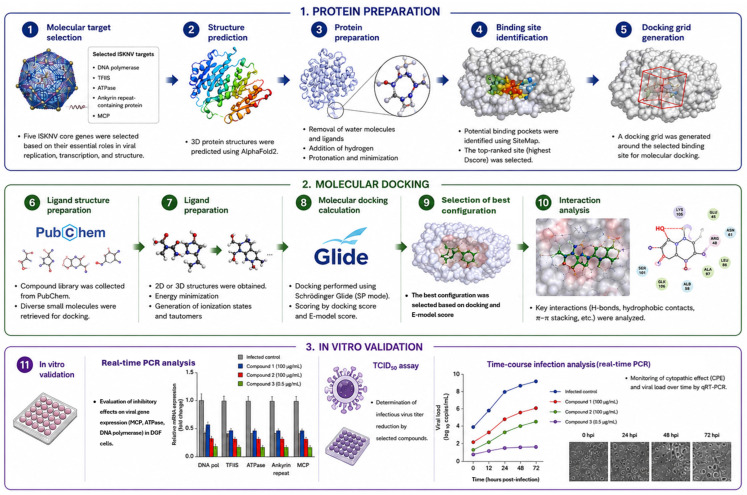
Schematic workflow of infectious spleen and kidney necrosis virus (ISKNV) target selection, protein structure prediction, molecular docking, candidate prioritization, and subsequent *in vitro* evaluation.

The Schrödinger Maestro suite, version 14.5 (Schrödinger, LLC, New York, NY, USA), was used for all the computational analyses. The workflow included the preparation of ligands and proteins, prediction of binding pockets using SiteMap, receptor grid generation with Glide, molecular docking, calculation of docking scores, and visualization of ligand–protein interactions. The experimental workflow used in this study is illustrated in Figure 1.

### 2.2. Ligand Preparation

A total of 72 compounds were selected to form ligand libraries for molecular docking analysis. Chemical information for each compound, including the compound name, PubChem Compound Identifier (CID), Chemical Abstracts Service (CAS) number, and molecular formula, was obtained from the PubChem database. The initial compound library was established through an extensive literature survey of compounds previously reported to exhibit antiviral activity against DNA and RNA viruses, including human and aquatic animal viruses. Candidate compounds were collected based on published evidence of antiviral efficacy or their potential for drug repurposing as antiviral agents, regardless of their original clinical indications. The resulting library comprised FDA-approved drugs, investigational or non-approved compounds, natural products, and other repurposed compounds, which were subsequently classified according to their pharmacological characteristics, biological applications, and regulatory status (Table 1). Chemical structures were imported into Schrödinger Maestro and prepared for molecular docking using the LigPrep module. During ligand preparation, three-dimensional structures were created, ionization states were assigned, and energy-minimized ligand conformations were produced for subsequent docking analysis.

### 2.3. Selection and Sequence Preparation of ISKNV Target Proteins

Viral target protein candidates were identified by examining the conserved core genes of ISKNV and their predicted functional relevance to viral replication, transcription, viral structure, and host interaction, using the reference genome sequence of ISKNV (GenBank accession no. AF371960). Based on this screening, five ISKNV proteins were selected for molecular docking studies: DNA polymerase, transcription elongation factor (TFIIS), adenosine triphosphatase (ATPase), ankyrin repeat-containing protein, and major capsid protein (MCP) (Table 2).

Primers were designed to amplify the complete ORFs of the selected genes from the ISKNV-SAY-23 isolate, which was detected in dwarf gourami imported from Singapore in 2023 [5]. Polymerase chain reaction (PCR)-amplified products were cloned using the T-Blunt™ PCR cloning kit (SolGent, Daejeon, Republic of Korea) according to the manufacturer’s instructions. The cloned inserts were subjected to Sanger sequencing, and the confirmed nucleotide sequences were translated into amino acid sequences for subsequent structure prediction and molecular docking studies. The primer sequences and expected product sizes for complete ORF amplification are listed in Table 3.

### 2.4. Protein Structure Prediction, Preparation, SiteMap Analysis, and Receptor Grid Generation

The amino acid sequences of the selected ISKNV proteins were used as input for three-dimensional structure prediction with ColabFold version 1.6.1 (AlphaFold2 using MMseqs2). The protein models predicted by AlphaFold were exported in the Protein Data Bank (PDB)-format files and imported into Schrödinger Maestro for protein preparation. The protein models were processed using the Protein Preparation Wizard, involving the assignment of bond orders, addition of hydrogen atoms, optimization of hydrogen-bonding networks, and restrained energy minimization.

Potential ligand-binding pockets were predicted using the SiteMap module of Schrödinger Maestro. The binding sites were evaluated based on pocket size, DScore, SiteScore, and pocket volume. For each target protein, docking grids were generated using the Glide receptor grid-generation module based on the predicted binding sites. For ATPase, two independent docking grids were generated because distinct SiteMap regions were selected: one grid included site 1, whereas the other grid included sites 2, 3, 4, and 5. SiteMap and grid selection criteria are listed in Table 4.

### 2.5. Molecular Docking and Interaction Analysis

Molecular docking was executed using the Schrödinger Maestro Glide module. Ligands, once prepared, were docked into the receptor grids of each selected ISKNV protein using the standard precision (SP) mode. The docking results were evaluated using Glide docking and E-model scores. The Glide docking score is an empirical scoring function used to estimate the relative favorability of ligand–protein interactions, whereas the E-model score is primarily used to rank ligand poses by combining the Glide docking score with energetic terms, including Coulombic, van der Waals, and ligand strain energies. Therefore, lower (more negative) docking scores indicate more favorable predicted ligand–protein interactions, and lower E-model scores indicate more favorable and stable predicted binding poses.

For each target protein, the docking scores were ranked to identify the highest-scoring compounds. The top 20 compounds for each of the five ISKNV target proteins were extracted and compared across all the targets. Compounds that consistently appeared in the top 20 list for all five target proteins were prioritized as candidate compounds for further *in vitro* evaluations. Based on these criteria, fludarabine, liquiritin, and lycorine were selected for assessment. For representative interaction analysis, the compound with the highest docking score among the three selected candidates for each target protein was visualized. Three-dimensional binding poses and two-dimensional ligand–protein interaction maps were generated using the Schrödinger Maestro software to identify key ligand–residue interactions within the predicted docking pockets.

### 2.6. Cell Culture and Compounds

DGF cells previously established in our laboratory were used for all the *in vitro* assays. The cells were maintained at 28 °C in Leibovitz’s medium (L-15; Gibco, Waltham, MA, USA) supplemented with 10% fetal bovine serum (FBS; Gibco, Waltham, MA, USA), non-essential amino acids (NEAA; Gibco, Waltham, MA, USA), and hydroxyethyl piperazine ethane sulfonic acid (HEPES; Gibco, Waltham, MA, USA), as previously described, with minor modifications [30].

Fludarabine and lycorine were purchased from MedChemExpress (MCE; Monmouth Junction, NJ, USA), and liquiritin was purchased from Sigma-Aldrich (St. Louis, MO, USA). Stock solutions (20 mg/mL) of each compound were prepared by dissolving 10 mg of compound in 500 μL of dimethyl sulfoxide (DMSO). These stock solutions were aliquoted and stored at −80 °C until use. For the *in vitro* experiments, stock solutions were diluted with L-15 medium to obtain the specified working concentrations. The final concentration of DMSO in the treated samples was adjusted to be less than 0.01%, which was confirmed to be non-toxic to DGF cells.

### 2.7. Cytotoxicity Assay

The cytotoxic effects of fludarabine, liquiritin, and lycorine on DGF cells were evaluated using the CCK-8 assay. Briefly, DGF cells were seeded in a monolayer in 96-well plates at a density of 4 × 10^4^ cells/well and incubated at 28 °C until cell attachment. The cells were exposed to different concentrations of each compound. After 72 h of treatment, the CCK-8 reagent (Sigma-Aldrich, St. Louis, MO, USA) was added to each well, and absorbance was measured at 450 nm using a microplate spectrophotometer (Mobi, MicroDigital Co., Ltd., Seongnam, Republic of Korea). Cell viability was calculated in comparison to the untreated control group. Dose–response curves were generated using nonlinear regression using a four-parameter logistic model in GraphPad Prism version 10.6. The concentrations for the fludarabine and liquiritin cytotoxicity tests were selected according to Baek et al. (2025) [31], and lycorine was used at concentrations below its cytotoxic range.

### 2.8. Analysis of Viral Gene Expression Following Compound Treatment

To evaluate the inhibitory effects of selected compounds on viral gene expression, DGF cells were seeded in 24-well plates at a density of 2 × 10^5^ cells/well and incubated until a confluent monolayer was formed. The cells were infected with ISKNV at 1 × 10^5^ median tissue culture infectious dose (TCID_50_) and incubated at 28 °C for 2 h to facilitate viral adsorption. After adsorption, unbound viral particles were removed, and the cells were rinsed twice with phosphate-buffered saline (PBS). Fresh L-15 medium with 5% FBS was then added, containing either fludarabine (100 μg/mL), liquiritin (100 μg/mL), or lycorine (0.5 μg/mL). The infected cells were incubated at 28 °C for 72 h. After 72 h post-infection, the culture supernatant was discarded, and the cells were washed twice with PBS. Total RNA was extracted using the yesR^TM^ Total RNA Extraction Kit (GenesGen, Busan, Republic of Korea) according to the manufacturer’s instructions. The concentration and purity of RNA were measured using a NanoVue Plus spectrophotometer (GE Healthcare, Chicago, IL, USA), and RNA samples with acceptable A260/A280 ratios were used for cDNA synthesis. cDNA was synthesized using a PrimeScript™ 1st cDNA Synthesis Kit (Takara, Shiga, Japan) according to the manufacturer’s instructions.

The mRNA expression levels of the ISKNV MCP, ATPase, and DNA polymerase genes were quantified through real-time PCR using 2 × qPCRBIO SyGreen Blue Mix Lo-ROX (PCR Biosystems Ltd., London, UK) in a qTower^3^ Real-Time Thermocycler (Analytik Jena GmbH, Jena, Germany). Each PCR included gene-specific forward and reverse primers at a final concentration of 1 µM. The real-time PCR conditions and primer sequences are presented in Table 5. The experiment was conducted in triplicate, using DGF cell *β*-actin as an endogenous control. Specificity was verified with a melt curve, and the relative transcription levels of the target gene were analyzed using the comparative Ct method (2^−ΔΔCt^) [32]. Finally, inhibition percentages were calculated as follows: Inhibition (%) = {1 − (2^−ΔΔCt^ of tested sample/2^−ΔΔCt^ of control sample)} × 100. Statistical analyses were performed using one-way analysis of variance (ANOVA) with GraphPad Prism version 10.6.

### 2.9. Determination of Viral Titers Following Compound Treatment

To determine the effects of the selected compounds on the production of infectious virus, viral titers were measured using the TCID_50_ assay [35]. DGF cells were seeded in 96-well plates at a density of 4 × 10^4^ cells/well and incubated until a monolayer formed. Culture supernatants containing ISKNV were serially diluted 10-fold and inoculated into DGF cells. After 2 h of adsorption at 28 °C, the inoculum was discarded, and the cells were washed twice with PBS to eliminate any unbound viral particles. For treatment, L-15 medium supplemented with 5% FBS and the test compound was used. The treatment concentrations were fludarabine at 100 μg/mL, liquiritin at 100 μg/mL, and lycorine at 0.5 μg/mL. At 10 days post-infection, CPE were observed, and viral titers were calculated as TCID_50_/mL.

### 2.10. Time-Course Analysis of Cytopathic Effects and Extracellular Viral Load

For time-course analysis, DGF cells were seeded in 24-well plates at a density of 2 × 10^5^ cells/well and incubated until a confluent monolayer was formed. The cells were infected with ISKNV at 1 × 10^5^ TCID_50_ and incubated at 28 °C for 2 h. After viral adsorption, the inoculum was removed, and the cells were washed twice with PBS. Fresh L-15 medium containing 5% FBS and each compound was added (fludarabine, 100 μg/mL; liquiritin, 100 μg/mL; and lycorine, 0.5 μg/mL). Cell morphology and CPE were monitored from 72 to 144 h post-infection using a microscope.

To quantify the extracellular viral load, culture supernatants were collected at 24, 48, 72, 96, 120, and 144 h post-infection. Viral DNA was extracted from the supernatant using the yesG^TM^ Cell Tissue Mini Kit (GenesGen, Busan, Republic of Korea). Real-time PCR targeting the ISKNV MCP gene was performed using a TaqMan probe-based assay, as described by Kim et al. (2021) [34]. Viral load was calculated based on the MCP gene copy number (Table 5), and log_10_-transformed values were used for subsequent statistical analyses.

## 3. Results

### 3.1. Selection of ISKNV Target Proteins and Molecular Docking Analysis

Five ISKNV viral proteins were selected as molecular docking targets based on their conserved core gene functions and predicted biological relevance to viral DNA replication, transcriptional regulation, virion assembly, host interaction, and viral structure (Table 3). Complete ORFs of the selected genes were amplified, cloned, and confirmed by sequencing. The confirmed nucleotide sequences were translated into amino acid sequences and used for downstream structural predictions and docking analyses (Table A1). Molecular docking was performed against the selected ISKNV target proteins using a library of 72 compounds, and docking and E-model scores were calculated for each ligand–protein complex (Table 6). For each target protein, the top 20 compounds based on docking scores were extracted and compared across the five target proteins. Fludarabine, liquiritin, and lycorine were the only compounds consistently included among the top 20 docking-ranked compounds across all five target proteins. These compounds were therefore selected for subsequent *in vitro* evaluation because they demonstrated broad predicted binding compatibility rather than target-specific affinity (Table 7).

### 3.2. Representative Ligand–Protein Interaction Analysis

**Figure 2:**
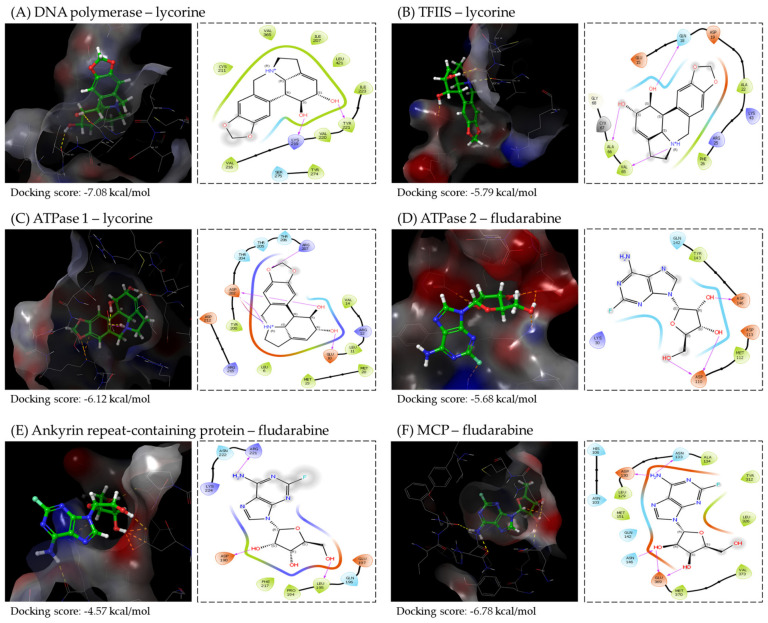
Molecular docking interactions between selected compounds and ISKNV target proteins. For each target protein, the highest-ranked compound among the three selected candidates was used for the interaction analysis. Three-dimensional binding poses and corresponding two-dimensional interaction maps were generated to visualize ligand-binding within the predicted docking pockets.

Representative ligand–protein interactions were visualized for each ISKNV target protein using the compound that showed the highest docking score among the three selected candidates. The complexes analyzed included DNA polymerase–lycorine, TFIIS–lycorine, ATPase 1–lycorine, ATPase 2–fludarabine, ankyrin repeat-containing protein–fludarabine, and MCP–fludarabine (Figure 2). The selected compounds were positioned within the predicted docking pockets and formed multiple interactions with surrounding amino acid residues. The three-dimensional docking poses showed that the ligands were accommodated within the protein surface cavities. In addition, two-dimensional interaction maps revealed hydrogen bonding, polar contacts, and hydrophobic interactions with residues located near the predicted binding sites, suggesting that the selected compounds have potential binding compatibility with multiple ISKNV target proteins. The principal hydrogen-bonding and hydrophobic interactions identified for the representative docking complexes are summarized in Table 8.

### 3.3. Cytotoxicity of Selected Compounds on DGF Cells

**Figure A1:**
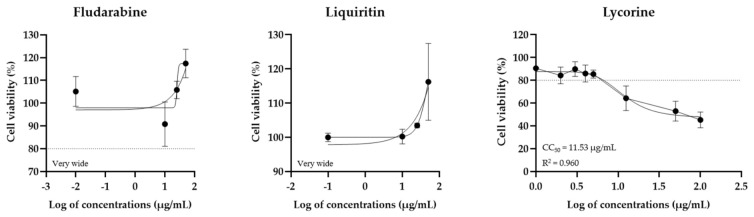
Cytotoxicity assessment of fludarabine, liquiritin, and lycorine in DGF cells. Cell viability was measured using the CCK-8 assay 72 h after treatment with the test compound, and absorbance was determined at 450 nm. Dose–response curves were generated using nonlinear regression with a four-parameter logistic model based on log-transformed concentrations in GraphPad Prism version 10.6. Data are presented as mean ± SD from three independent experiments.

The treatment concentrations of fludarabine, liquiritin, and lycorine were determined using previous cytotoxicity data and further cytotoxicity evaluations in DGF cells (Figure A1). Based on the cytotoxicity findings from our previous study [31], fludarabine and liquiritin were used at a concentration of 100 μg/mL for subsequent antiviral testing. The cytotoxicity of lycorine was evaluated in DGF cells using a CCK-8 assay after 72 h of exposure to the compound. Lycorine exhibited concentration-dependent cytotoxicity, with a calculated CC_50_ value of 11.53 μg/mL. Although the 72 h cytotoxicity assay showed detectable cell viability at concentrations exceeding 0.5 μg/mL, extended incubation during the infection time-course analysis showed morphological changes at 1–10 μg/mL concentrations. Therefore, lycorine was used at 0.5 μg/mL to minimize cytotoxic effects during extended antiviral evaluation.

### 3.4. Antiviral Effects of Selected Compounds on ISKNV Viral Gene Expression

**Figure 3:**
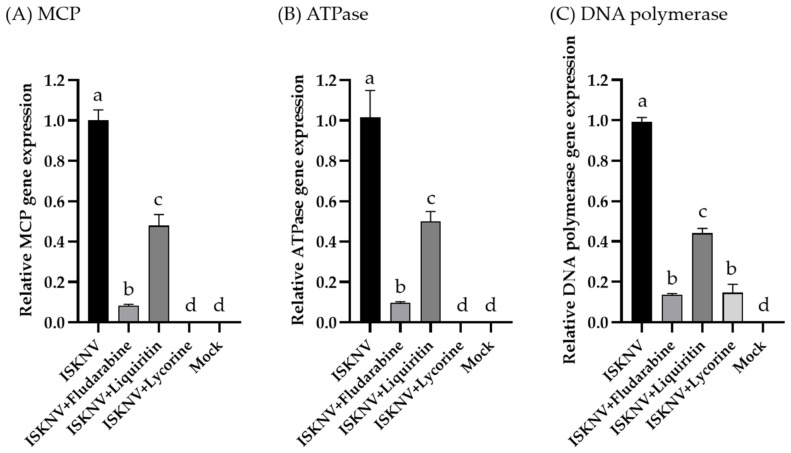
Relative mRNA expression levels of MCP, ATPase, and DNA polymerase in ISKNV-infected DGF cells 72 h post-infection. Expression levels were quantified by real-time PCR and normalized to *β*-actin. Data are presented as the mean ± SD from three independent experiments. Statistical analysis was performed using one-way analysis of variance (ANOVA) followed by Tukey’s multiple comparison test in GraphPad Prism version 10.6. Different letters indicate statistically significant differences among groups (*p* < 0.05).

To assess the antiviral effects of the selected compounds on viral gene expression, ISKNV-infected DGF cells were treated with fludarabine, liquiritin, or lycorine, and the relative mRNA expression levels of MCP, ATPase, and DNA polymerase were analyzed 72 h post-infection using real-time PCR. All three compounds reduced the expression levels of ISKNV target genes to varying degrees compared to the infected control (*p* > 0.05; Figure 3). The calculated inhibition rates are listed in Table 9. In this study, inhibition rates greater than 80% were considered to indicate strong inhibitory effects. Fludarabine showed strong inhibition of all three viral genes, with inhibition rates of 91.67 ± 0.72%, 90.66 ± 0.35%, and 86.11 ± 0.82% for MCP, ATPase, and DNA polymerase, respectively. Liquiritin exhibited moderate inhibitory effects, with inhibition rates of 52.11 ± 5.60%, 50.33 ± 5.16%, and 56.41 ± 2.59% for MCP, ATPase, and DNA polymerase, respectively. Lycorine also showed strong inhibition of MCP, ATPase, and DNA polymerase expression, with inhibition rates of 99.81 ± 0.08%, 99.66 ± 0.09%, and 85.46 ± 4.33%, respectively.

### 3.5. Effects of Treatments on Infectious ISKNV Production

**Figure 4:**
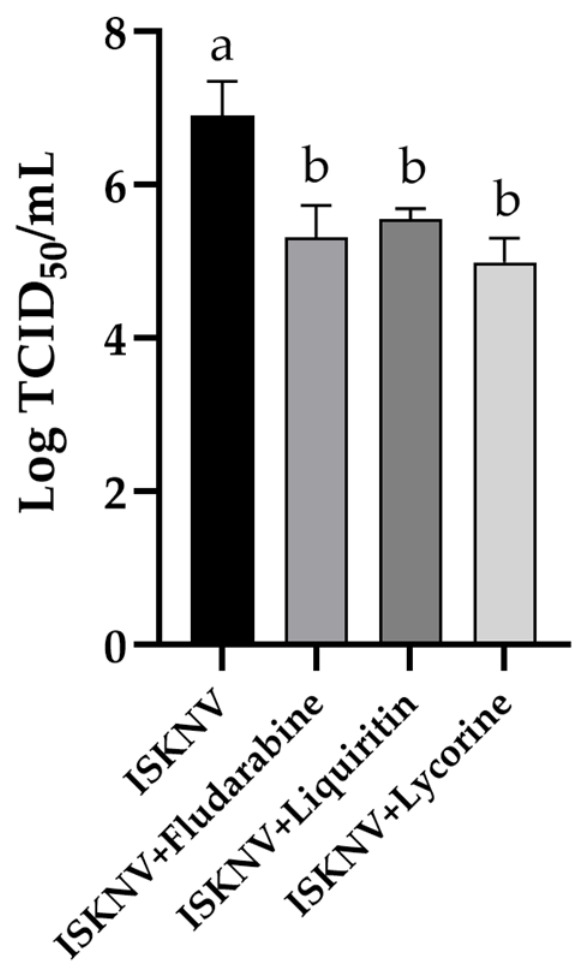
Infectious ISKNV titers in DGF cells following treatment with selected compounds at single concentrations. Viral titers were measured using the TCID_50_ assay following treatment with fludarabine (100 μg/mL), liquiritin (100 μg/mL), and lycorine (0.5 μg/mL). Data are presented as mean ± SD from three independent experiments. Statistical analysis was performed using one-way ANOVA followed by Tukey’s multiple comparison test. Statistically significant differences between groups are indicated by different letters (*p* < 0.05).

To determine whether the selected compounds affected infectious virus production, extracellular viral titers were measured using the TCID_50_ assay after applying the test compound treatment. Across three independent experiments, the ISKNV-infected control group exhibited an infectious viral titer of 6.90 ± 0.44 log_10_ TCID_50_/mL. The viral titers of the fludarabine-, liquiritin-, and lycorine-treated groups were 5.31 ± 0.42, 5.55 ± 0.14, and 4.99 ± 0.31 log_10_ TCID_50_/mL, respectively (Figure 4). Compared with the infected control group, the treatments with fludarabine, liquiritin, and lycorine resulted in reduced extracellular infectious viral titers by approximately 39-fold, 22-fold, and 81-fold, respectively. Among the compounds tested, lycorine was the most effective in decreasing infectious viral titers under the test conditions.

### 3.6. Time-Course of CPE Progression

**Figure 5:**
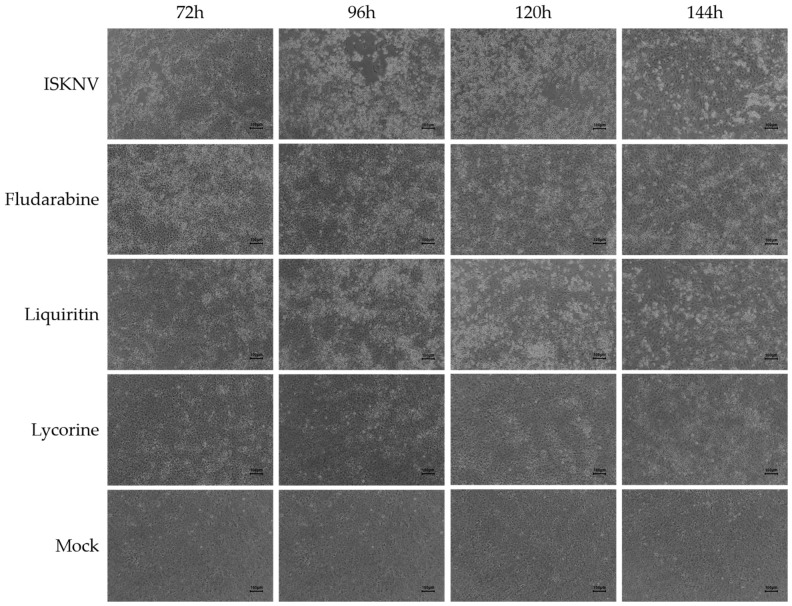
Microscopic observations over time of cytopathic effects (CPE) in ISKNV-infected DGF cells after treatment with selected compounds. DGF cells were infected with ISKNV and treated with fludarabine (100 μg/mL), liquiritin (100 μg/mL), or lycorine (0.5 μg/mL). Images of cell morphology were captured at specified intervals following infection. The progression of ISKNV-associated CPE, including cell enlargement, cell rounding, and monolayer destruction, was compared among the treatment groups. Scale bars = 100 μm.

Microscopic observations over time were conducted to compare CPE progression in ISKNV-infected DGF cells after treatment with the chosen compounds (Figure 5). Up to 48 h post-infection, no marked CPE was observed. In the ISKNV-infected control group, cell rounding, cellular enlargement, and monolayer disruption were evident at 72 h post-infection and progressively aggravated until 144 h post-infection. Conversely, the fludarabine-treated group maintained the cell monolayer up to 144 h post-infection, although some cells appeared enlarged and condensed. The liquiritin-treated cells exhibited cellular shrinkage and enlargement at 96 h post-infection, with partial monolayer disruption at 120 h post-infection, and at 144 h post-infection, the CPE pattern resembled that of the infected control group. The lycorine-treated cells showed only mild cellular enlargement, and their overall cell morphology remained comparable to that of mock cells throughout the observation period.

### 3.7. Time-Course Analysis of Extracellular Viral Load

**Figure 6:**
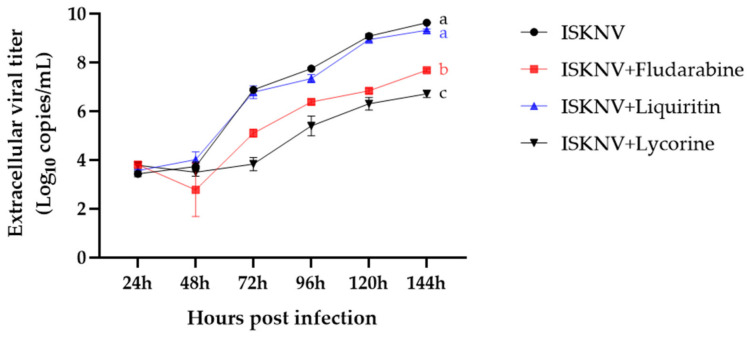
Time-course analysis of extracellular ISKNV viral load in DGF cells treated with specific compounds. Viral load was quantified by MCP-targeted real-time PCR using culture supernatants collected at specified time points and expressed as log_10_ copies/mL. Data are presented as mean ± SD from three independent experiments. Statistical analysis was performed using two-way ANOVA, followed by Tukey’s multiple comparison test in GraphPad Prism version 10.6. Different letters indicate statistically significant differences among the groups at each time point (*p* < 0.05).

The extracellular viral load was quantified in the culture supernatants collected at various time intervals, using real-time PCR targeting the ISKNV MCP gene (Figure 6). Viral load increased progressively in the ISKNV-infected control group from 3.43 ± 0.12 log_10_ copies at 24 h post-infection to 9.63 ± 0.01 log_10_ copies/mL at 144 h post-infection. Fludarabine treatment suppressed the rise in extracellular viral load at 72 h post-infection, resulting in 5.10 ± 0.17, 6.38 ± 0.11, 6.84 ± 0.11, and 7.68 ± 0.07 log_10_ copies/mL at 72, 96, 120, and 144 h post-infection, respectively. Lycorine exhibited the most potent inhibitory effect, with viral loads of 3.83 ± 0.27, 5.39 ± 0.40, 6.30 ± 0.26, and 6.71 ± 0.14 log_10_ copies/mL at the corresponding time points. The liquiritin-treated cells exhibited viral loads comparable to those of the infected control group, particularly at later time points, reaching 9.32 ± 0.05 log_10_ copies/mL at 144 h post-infection. Overall, fludarabine and lycorine reduced extracellular ISKNV release over time, whereas liquiritin demonstrated limited inhibition during extended periods of infection. Statistical analysis using log_10_-transformed viral load values revealed significant differences between the fludarabine- and lycorine-treated groups and the ISKNV-infected control group at 144 h post-infection (*p* < 0.05), whereas the liquiritin-treated group did not differ significantly from the infected control group (*p* > 0.05).

## 4. Discussion

Structure-based molecular docking is frequently used as an initial screening approach for discovering antiviral candidates because it allows rapid prioritization of compounds predicted to interact with viral target proteins [19,21]. Recent studies have demonstrated that virtual screening approaches can efficiently narrow down the number of candidate compounds before biological validation, thus enhancing the efficiency of antiviral drug discovery processes [27,36]. However, several studies have indicated that docking scores do not always directly correlate with actual biological or antiviral efficacy, suggesting that computational prediction alone may not fully account for antiviral activity [27]. In the present study, a multi-target docking strategy was applied to reduce the potential bias associated with single-target screening. Instead of selecting only the top-ranked compound for each target, compounds that consistently ranked among the top candidates across multiple viral proteins were prioritized. This strategy was intended to identify compounds with potential multi-target interactions, which may provide broader antiviral activity against ISKNV while reducing dependence on inhibition of a single viral protein. This approach may enhance the reliability of candidate selection by identifying compounds with broadly predicted interactions involving multiple viral processes. Consistent with the docking results, fludarabine and lycorine showed relatively significant antiviral activity *in vitro*, suggesting that repeated high-ranking interactions across multiple viral proteins are associated with their antiviral potential against ISKNV.

Among the tested compounds, lycorine demonstrated the strongest antiviral effect against ISKNV *in vitro*. Lycorine is a natural alkaloid isolated from Amaryllidaceae plants that exhibits broad-spectrum antiviral activity against several RNA and DNA viruses, including coronaviruses, enterovirus 71, chikungunya virus, dengue virus, and herpes simplex virus [37,38,39,40]. Previous studies have shown that lycorine inhibits viral replication by decreasing viral RNA levels and suppressing genome replication across different viral systems [38,39]. Consistent with these reports, lycorine significantly suppressed the expression of MCP, ATPase, and DNA polymerase genes. The coordinated suppression of three representative viral genes was accompanied by reductions in infectious viral titers and extracellular viral load, as well as delayed CPE progression, suggesting that lycorine affected multiple ISKNV replication-associated endpoints. Notably, lycorine achieved the most substantial reduction in infectious viral titer among the compounds tested, indicating that the observed transcriptional suppression was translated into reduced productive viral replication. Although direct inhibition of the predicted target proteins was not experimentally confirmed, the overall consistency between the docking predictions and the *in vitro* findings supports the antiviral potential of lycorine against ISKNV.

Fludarabine also demonstrated significant antiviral activity in this study. Fludarabine is a purine nucleoside analog that has been clinically used to treat hematological malignancies and is known to interfere with nucleic acid synthesis by inhibiting DNA polymerase activity and chain elongation [41,42,43]. In this study, fludarabine reduced viral gene expression and infectious viral titers, consistent with its predicted mechanism of interfering with DNA synthesis. ISKNV is a large double-stranded DNA virus that depends on viral DNA replication for successful infection. Therefore, the antiviral activity of fludarabine may be associated with interference with viral DNA synthesis-related processes.

Liquiritin moderately inhibited viral gene expression during the early infection stages; however, its inhibitory effect was less apparent during the extended time-course analysis. Liquiritin, a flavonoid derived from licorice, is recognized for its antiviral [44], antioxidant [45], and anti-inflammatory activities [46]. Although the exact mechanism by which liquiritin acts against ISKNV remains unclear, the current findings suggest that its antiviral effect under the conditions tested may be relatively weaker than that of fludarabine and lycorine.

Although all three selected compounds reduced ISKNV gene expression at 72 h post-infection, their effects on infectious virus production and long-term CPE progression differed. This variation can be attributed to the distinct biological information provided by each assay. Real-time PCR measures changes in viral transcript or genome copy numbers, whereas TCID_50_ and CPE observations indicate the generation of infectious progeny viruses and the resulting virus-induced cellular damage. Therefore, reduced viral mRNA expression at a single time point does not necessarily indicate the sustained inhibition of viral protein synthesis, virion assembly, or infectious particle production. In this study, liquiritin moderately reduced MCP, ATPase, and DNA polymerase expression at 72 h post-infection, but the extracellular viral load and CPE progression at later time points were comparable to those of the ISKNV-infected control group. This suggests that liquiritin may transiently inhibit viral gene expression, which may be insufficient to block downstream viral replication events, leading to infectious virus release under the present experimental conditions. Previous studies have demonstrated that mRNA levels do not always correlate directly with protein expression levels, particularly in dynamic biological contexts involving post-transcriptional regulation and protein turnover [47,48]. Conversely, fludarabine and lycorine exhibited consistent inhibition of viral gene expression, infectious titer, extracellular viral load, and CPE progression, supporting their potential antiviral activity against ISKNV. Although these findings do not conclusively prove the enzymatic inhibition of these proteins, they imply that the selected compounds might disrupt ISKNV replication through multi-target effects on replication-associated and structural viral components.

This study highlights the effectiveness of molecular docking as a preliminary screening tool for selecting antiviral candidates against aquatic animal viruses. Experimental antiviral screening using fish cell lines and large DNA viruses often requires extended incubation periods, cytotoxicity evaluations, viral gene quantification, and infectivity assays, making large-scale compound screening labor-intensive and time-consuming. Structure-based docking can streamline this process and reduce the number of compounds subjected to downstream biological assays by prioritizing candidates with predicted interactions with functionally relevant viral proteins. In the present study, the docking-based selection strategy successfully narrowed the 72 compounds to three candidates, two of which, fludarabine and lycorine, exhibited consistent antiviral effects across molecular, infectivity-based, and morphological evaluations. These findings support the use of docking as a practical prescreening tool when combined with biological validation.

Despite these insights, this study has certain limitations. Molecular docking predicts potential ligand–protein interactions based on structural modeling; however, direct biochemical validation of target binding was not performed. Additionally, the antiviral evaluation was limited to a single fish cell line and a narrow range of compound concentrations under *in vitro* conditions. Although fludarabine and lycorine significantly reduced viral gene expression, infectious viral titers, and delayed CPE progression, the reduction in extracellular viral load was relatively limited, indicating that the selected compounds did not completely suppress ISKNV replication under the experimental conditions used in this study. These findings suggest that the observed antiviral effects were partial and should be interpreted cautiously. Furthermore, this study focused on compounds that consistently exhibited favorable docking performance across multiple viral targets. Further studies comparing this multi-target prioritization strategy with compounds showing exceptionally high affinities for individual viral targets would provide further insight into the relative advantages of these screening approaches. In addition, incorporating established antiviral compounds with known activity against ISKNV and compounds with limited or no antiviral activity would provide valuable benchmarks for further validating the predictive performance of docking-based screening strategies. Therefore, additional studies, including dose-dependent antiviral investigations, molecular mechanisms, protein-level validations, and *in vivo* challenge experiments, are required to further evaluate the antiviral potential of the selected compounds against ISKNV. Nevertheless, the present study demonstrated that a multi-target docking-based screening strategy combined with biological validation can be effectively applied to identify potential antiviral candidates against ISKNV. Among the compounds tested, lycorine and fludarabine exhibited measurable antiviral activities and represent candidates for further investigation against ISKNV infection.

## 5. Conclusions

In conclusion, this study demonstrates the applicability of integrating multi-target molecular docking with comprehensive *in vitro* biological validation as a screening strategy for identifying antiviral candidates against ISKNV. Fludarabine and lycorine demonstrated consistent antiviral effects in alignment with docking-based prioritization and *in vitro* evaluation, whereas liquiritin exhibited weaker and less sustained antiviral activity, despite its favorable docking profile. Additionally, the reduction in extracellular viral load was limited, indicating that these compounds did not completely suppress ISKNV replication under the present experimental conditions. Therefore, fludarabine and lycorine should be considered preliminary antiviral candidates requiring further optimization and validation. Overall, these findings support the use of docking as a prioritization tool, while emphasizing that computational predictions should be interpreted within the limitations of structure-based modeling and confirmed through biological validation.

## Figures and Tables

**Table 1:** Antiviral compounds used for molecular docking analysis against selected ISKNV target proteins. Compounds were collected through literature reports describing antimicrobial activity and classified according to their reported pharmacological or chemical characteristics. PubChem compound identifier (CID) and molecular formulas were provided for compound identification and reproducibility.

No.	Compound Category	Compound Name	PubChem CID	CAS No.	Molecular Formula	Biological Use
1	FDA-approved chemical compound	5-Fluorouracil	3385	51-21-8	C_4_H_3_FN_2_O_2_	Anti-cancer
2		Abacavir sulfate	441384	188062-50-2	C_28_H_38_N_12_O_6_S	HIV
3		Adefovir dipivoxil	60871	142340-99-6	C_20_H_32_N_5_O_8_P	HBV
4		Aluminum hydroxide	10176082	21645-51-2	AlH_3_O_3_	Antacid/Vaccine adjuvant
5		Amantadine	2130	768-94-5	C_10_H_17_N	Influenza/Antiviral
6		Amodiaquine	2165	86-42-0	C_20_H_22_ClN_3_O	Malaria
7		Amprenavir	65016	161814-49-9	C_25_H_35_N_3_O_6_S	HIV protease inhibitor
8		Atovaquone	74989	94015-53-9	C_22_H_19_ClO_3_	Malaria/Antiparasitic
9		Atovaquone/proguanil	67439664	156879-69-5	C_33_H_36_Cl_3_N_5_O_3_	Malaria
10		Baloxavir marboxil	124081896	1985606-14-1	C_27_H_23_F_2_N_3_O_7_S	Influenza
11		Bictegravir	90311989	1611493-60-7	C_21_H_18_F_3_N_3_O_5_	HIV integrase inhibitor
12		Chloroquine	2719	54-05-7	C_18_H_26_ClN_3_	Malaria
13		Cytarabine	6253	147-94-4	C_9_H_13_N_3_O_5_	Anticancer
14		Darunavir	213039	206361-99-1	C_27_H_37_N_3_O_7_S	HIV protease inhibitor
15		Didanosine	135398739	69655-05-6	C_10_H_12_N_4_O_3_	HIV
16		Dolutegravir	54726191	1051375-16-6	C_20_H_19_F_2_N_3_O_5_	HIV integrase inhibitor
17		Emtricitabine	60877	143491-57-0	C_8_H_10_FN_3_O_3_S	HIV/HBV
18		Famciclovir	3324	104227-87-4	C_14_H_19_N_5_O_4_	Herpesvirus
19		Fludarabine	657237	21679-14-1	C_10_H_12_FN_5_O_4_	Anticancer
20		Ganciclovir	135398740	82410-32-0	C_9_H_13_N_5_O_4_	CMV/Herpesvirus
21		Halofantrine	37393	69756-53-2	C_26_H_30_Cl_2_F_3_NO	Malaria
22		Lamivudine	60825	134678-17-4	C_8_H_11_N_3_O_3_S	HIV/HBV
23		Lopinavir	92727	192725-17-0	C_37_H_48_N_4_O_5_	HIV protease inhibitor
24		Lumefantrine	6437380	82186-77-4	C_30_H_32_Cl_3_NO	Malaria
25		Maraviroc	483925835	376348-65-1	C_29_H_41_F_2_N_5_O	HIV entry inhibitor
26		Mefloquine	40692	51742-87-1	C_17_H_16_F_6_N_2_O	Malaria
27		Oseltamivir	65028	196618-13-0	C_16_H_28_N_2_O_4_	Influenza
28		Primaquine	4908	90-34-6	C_15_H_21_N_3_O	Malaria
29		Proguanil	6178111	500-92-5	C_11_H_16_ClN_5_	Malaria
30		Sofosbuvir	45375808	1190307-88-0	C_22_H_29_FN_3_O_9_P	HCV
31		Sulfadoxine	17134	2447-57-6	C_12_H_14_N_4_O_4_S	Malaria
32		Tecovirimat	16124688	869572-92-9	C_19_H_15_F_3_N_2_O_3_	Orthopoxvirus/Smallpox
33		Tenofovir	464205	147127-20-6	C_9_H_14_N_5_O_4_P	HIV/HBV
34		Zalcitabine	24066	7481-89-2	C_9_H_13_N_3_O_3_	HIV
35		Zidovudine	35370	30516-87-1	C_10_H_13_N_5_O_4_	HIV
36	Non-FDA- approved chemical compound	MLN4924 (Pevonedistat)	16720766	905579-51-3	C_21_H_25_N_5_O_4_S	Experimental anticancer compound
37		N-acetylcysteine amide	10176265	38520-57-9	C_5_H_10_N_2_O_2_S	Antioxidant derivative compound
38		Ritiometan	65787	34914-39-1	C_7_H_10_O_6_S_3_	Experimental compound
39		Rupintrivir	6440352	223537-30-2	C_31_H_39_FN_4_O_7_	Experimental antiviral compound
40	FDA-approved natural product-derived compound	Artemether	68911	71963-77-4	C_16_H_26_O_5_	Malaria
41		Artemisinin	68827	63968-64-9	C_15_H_22_O_5_	Malaria
42		Artenimol	3000518	71939-50-9	C_15_H_24_O_5_	Malaria
43		Artesunate	6917864	88495-63-0	C_19_H_28_O_8_	Malaria
44		Quinine	3034034	130-95-0	C_20_H_24_N_2_O_2_	Malaria
45		Squalene	638072	111-02-4	C_30_H_50_	Vaccine adjuvant/Natural lipid
46	Non-FDA- approved natural product-derived compound	(−)-Epigallocatechin gallate	65064	989-51-5	C_22_H_18_O_11_	Polyphenol/Green tea-derived compound
47		24-Ethylcholest-5-en-3beta-ol	22012	5779-62-4	C_29_H_50_O	Phytosterol
48		Andrograpanin	11666871	82209-74-3	C_20_H_30_O_3_	Andrographis-derived diterpenoid
49		Andrographiside	44593583	82209-76-5	C_26_H_40_O_10_	Andrographis-derived compound
50		Apigetrin (Cosmosiin)	5280704	578-74-5	C_21_H_20_O_10_	Flavonoid glycoside
51		Arctigenin	64981	7770-78-7	C_21_H_24_O_6_	Lignan phytochemical
52		Baicalin	64982	21967-41-9	C_21_H_18_O_11_	Flavonoid
53		Berberine	2353	2086-83-1	C_20_H_18_NO_4_^+^	Isoquinoline alkaloid
54		Betulonal	473111	4439-98-9	C_30_H_46_O_2_	Triterpenoid
55		Cerevisterol	10181133	516-37-0	C_28_H_46_O_3_	Sterol
56		Chrysin	5281607	480-40-0	C_15_H_10_O_4_	Flavonoid
57		Clivimine	44559309	7096-85-7	C_43_H_43_N_3_O_12_	Alkaloid
58		Ethyl ferulate	736681	4046-02-0	C_12_H_14_O_4_	Phenolic ester
59		Ferulic acid	445858	1135-24-6	C_10_H_10_O_4_	Phenolic compound
60		Fucoidan from *Fucus vesiculosus*	92023653	9072-19-9	C_7_H_14_O_7_S	Marine polysaccharide
61		Gnidicin	70691946	55319-39-6	C_36_H_36_O_10_	Natural product
62		Hesperidin	10621	520-26-3	C_28_H_34_O_15_	Flavonoid glycoside
63		Honokiol	72303	35354-74-6	C_18_H_18_O_2_	Lignan
64		Kaempferol	5280863	520-18-3	C_15_H_10_O_6_	Flavonoid
65		Liquiritin	503737	551-15-5	C_21_H_22_O_9_	Flavonoid glycoside
66		Lycorine	72378	476-28-8	C_16_H_17_NO_4_	Alkaloid
67		Neohesperidin	442439	13241-33-3	C_28_H_34_O_15_	Flavonoid glycoside
68		Piceatannol	667639	10083-24-6	C_14_H_12_O_4_	Polyphenol
69		Rosmarinic acid	5281792	20283-92-5	C_18_H_16_O_8_	Phenolic compound
70		Theaflavine-3,3′-digallate-3,3′-digallate	21146795	30462-35-2	C_43_H_32_O_20_	Tea polyphenol
71		Ursolic acid	64945	77-52-1	C_30_H_48_O_3_	Triterpenoid
72		α-Mangostin	5281650	6147-11-1	C_24_H_26_O_6_	Xanthone phytochemical

**Table 2:** Functional classification and rationale for selection of ISKNV proteins used for molecular docking analysis based on the reference genome AF371960.

No.	Target Protein	ORF No. (AF371960)	Functional Category	Predicted/Known Function	Reference
1	DNA polymerase	ORF019R	Viral DNA replication	Catalyzes viral genome replication and is associated with DNA synthesis and proofreading	[1,28]
2	TFIIS	ORF029L	Transcription regulation	Facilitates transcription elongation and maintains RNA polymerase fidelity	[1,28]
3	ATPase	ORF122R	Viral replication/ Virion assembly	Provides energy for viral replication, DNA packaging, or virion assembly through ATP hydrolysis	[1,28]
4	Ankyrin repeat- containing protein	ORF125L	Host interaction/ Immune modulation	Mediates protein–protein interactions involved in host signaling and immune modulation	[1,29]
5	MCP	ORF006L	Viral structure	Essential structural protein forming the viral capsid and maintaining virion stability	[1,28]

**Table 3:** Primers used to amplify complete open reading frames (ORFs) of selected ISKNV genes.

Target Gene	ORF No. (AF371960)	Sequence (5′–3′)	Tm (°C)	Product Size (bp)
DNA polymerase	ORF019R	F: ATG GAT AGT GTG TAC ATC TAT CAG TGG CTC	60	2847
		R: TCA TAC GGC AGG CGT CGT GCG TCT CAG TAA		
TFIIS	ORF029L	F: ATG TAT ACA TGT ACA	60	222
		R: TCA AAT GCG ATA GCG		
ATPase	ORF122R	F: ATG GAA ATC AAA GAG TTG TCC TTG ACG	60	720
		R: TTA CGC CAC GCC AGC CTT		
Ankyrin repeat- containing protein	ORF125L	F: ATG CTG CCC GAG GAG CTT	60	687
		R: TTA GGC GAA AAA GTC TTT ATT GTT CCG GG		
MCP	ORF006L	F: ATG TCT GCA ATC TCA G	60	1362
		R: TTA CAG GAT AGG GAA G		

**Table 4:** SiteMap analysis and binding pocket prediction of selected ISKNV proteins used for molecular docking. SiteScore and DScore values were used to identify potential ligand-binding sites for grid generation.

Target Protein	Site No.	Size	DScore	SiteScore	Volume	Included Sites Within Docking Grid	Pocket Volume
DNA polymerase	site 1	157	1.070	1.025	564.578	1, 4	20
	site 2	79	0.891	0.976	178.017		
	site 3	73	0.902	0.888	197.225		
	site 4	45	0.657	0.762	81.977		
	site 5	26	0.509	0.592	80.605		
TFIIS	site 1	14	0.444	0.500	65.513	1	10
ATPase	site 1	70	0.766	0.864	213.346	1	15
	site 2	54	0.505	0.81	141.659	2, 3, 4, 5	20
	site 3	33	0.563	0.598	57.624		
	site 4	22	0.519	0.58	49.392		
	site 5	26	0.469	0.552	67.914		
Ankyrin repeat- containing protein	site 1	85	0.982	0.931	244.802	1, 3	20
	site 2	36	0.613	0.660	91.924		
	site 3	35	0.414	0.648	79.576		
	site 4	22	0.451	0.542	55.566		
MCP	site 1	124	1.052	1.047	315.56	1, 2, 5	20
	site 2	59	0.847	0.87	199.283		
	site 3	41	0.641	0.824	73.059		
	site 4	33	0.691	0.723	128.625		
	site 5	36	0.588	0.642	102.557		

**Table 5:** Primers and real-time PCR conditions used for *in vitro* evaluation of ISKNV gene expression.

Target Gene	Purpose	Sequence (5′–3′)	Conditions	Reference
MCP	mRNA expression	F: GGC GAC TAC CTC ATT AAT GT	95 °C, 10 min; (95 °C, 20 s; 52 °C, 1 min) × 40	[33]
		R: CCA CCA GGT CGT TAA ATG A		
ATPase	mRNA expression	F: ATA ATT CCC GCG GCC GTC	95 °C, 10 min; (95 °C, 20 s; 60 °C, 1 min) × 40	[31]
		R: CTC GGG GTC CAC GTT CTT		
DNA polymerase	mRNA expression	F: GTT TAT GGC GGG GGC AAT		[30]
		R: TGG CCC AGC TGT ATG TAG C		
*β*-actin	mRNA expression	F: TAG CCA CGC TCT GTC AGG AT		[30]
		R: ACC ACC GGT ATT GTC ATG GA		
MCP	Viral quantification	F: CCA GCA TGC CTG AGA TGG A	95 °C, 10 min; (94 °C, 10 s; 60 °C, 35 s) × 40	[34]
		R: GTC CGA CAC CTT ACA TGA CAG G		
		P: FAM-TAC GGC CGC CTG TCC AAC G-BHQ1		

**Table 6:** Docking and E-model scores of 72 antiviral-related compounds against five selected ISKNV target proteins. Lower docking and E-model scores indicate more favorable predicted binding affinity and ligand–protein interaction stability. Color gradients were applied to the docking scores for each target protein column to facilitate visual comparison, with red indicating lower (more favorable) docking scores and green indicating higher (less favorable) docking scores.

Compound Name	DNA Polymerase		TFIIS		ATPase 1		ATPase 2		Ankyrin Repeat- Containing Protein		MCP	
	Docking	E-Model	Docking	E-Model	Docking	E-Model	Docking	E-Model	Docking	E-Model	Docking	E-Model
5-Fluorouracil	−6.26	−29.26	−5.12	−27.44	−4.13	−26.35	−3.87	−39.93	−4.47	−27.91	−6.19	−35.56
Abacavir sulfate	−5.62	−44.74	−4.65	−38.68	−4.58	−40.52	−4.09	−36.06	−3.58	−37.82	−5.61	−50.35
Adefovir Dipivoxil	−3.62	−35.62	-	-	−3.33	−49.58	−3.47	−49.99	−2.07	−41.89	−5.62	−69.31
Amantadine	−4.11	−20.35	−4.44	−23.86	−3.65	−21.55	-	-	−3.72	−21.97	−5.52	−35.86
Amodiaquine	−6.43	−60.62	−4.48	−44.79	−4.38	−49.69	−4.03	−51.37	−3.73	−47.75	−4.34	−54.05
Amprenavir	−6.02	−51.69	−3.14	−34.24	−3.32	−39.23	−3.11	−38.68	−2.47	−35.11	−4.88	−52.25
Atovaquone	−5.66	−43.69	−4.37	−38.93	−2.95	−33.16	−2.9	−29.8	−2.10	−17.93	−4.24	−36.95
Atovaquone/proguanil	−5.66	−43.69	−4.37	−38.93	−2.95	−33.16	−2.9	−29.8	−2.10	−17.93	−4.24	−36.95
Baloxavir Marboxil	−4.80	−46.44	−3.57	−39.63	−3.49	−43.29	−2.22	−31.33	−2.56	−39.26	−3.70	−53.30
Bictegravir	−5.55	−53.38	−3.91	−45.98	−3.10	−38.34	−3.28	−49.84	−3.23	−41.12	−5.68	−61.81
Chloroquine	−3.91	−30.27	−4.10	−31.22	−4.39	−38.37	−3.73	−43.67	−4.26	−43.49	−6.27	−57.05
Cytarabine	−5.77	−41.45	−5.06	−38.40	−6.08	−49.99	−4.93	−41.00	−4.55	−34.72	−7.97	−59.00
Darunavir	−6.61	−61.67	−3.89	−38.40	−3.41	−39.11	−3.13	−38.82	−3.00	−40.96	−4.40	−46.90
Didanosine	−4.81	−42.29	−4.69	−35.13	−5.84	−47.79	−4.27	−38.27	−3.79	−30.30	−7.60	−56.28
Dolutegravir	−5.41	−44.82	−4.13	−44.87	−4.24	−51.18	−3.09	−41.27	−3.78	−47.56	−5.83	−55.86
Emtricitabine	−5.73	−38.19	−4.73	−27.62	−5.66	−48.88	−3.88	−33.03	−3.34	−29.62	−7.34	−49.88
Famciclovir	−4.68	−38.69	−3.64	−32.15	−4.22	−45.98	−2.98	−33.96	−2.81	−38.25	−5.45	−57.53
Fludarabine	−6.48	−46.42	−5.11	−39.17	−5.48	−49.01	−5.68	−52.06	−4.57	−34.15	−6.78	−52.95
Ganciclovir	−4.85	−45.72	−4.84	−41.01	−5.30	−52.14	−3.77	−41.39	−3.66	−34.54	−7.11	−63.73
Halofantrine	−6.71	−57.80	−3.17	−31.59	−3.62	−44.74	−3.09	−46.69	−3.12	−35.68	−4.54	−54.88
Lamivudine	−5.81	−42.32	−4.89	−33.87	−5.68	−46.59	−3.90	−32.84	−4.65	−31.59	−7.43	−53.79
Lopinavir	−6.59	−67.19	-	-	−3.79	−44.08	−4.18	−61.16	−4.50	−56.79	−7.17	−84.91
Lumefantrine	−5.99	−54.72	−3.95	−38.06	−2.66	−37.29	−2.42	−39.43	−2.46	−38.26	−5.00	−62.23
Maraviroc	−5.25	−41.50	−3.06	−29.41	−4.13	−49.67	−3.50	−45.80	−3.32	−33.78	−4.62	−53.33
Mefloquine	−7.36	−50.87	−4.76	−36.9	−5.06	−48.34	−4.05	−44.54	−4.41	−35.93	−4.61	−44.88
Oseltamivir	−4.63	−34.97	−4.86	−39.64	−4.36	−40.03	−3.97	−40.58	−3.20	−38.37	−4.90	−36.24
Primaquine	−6.34	−51.88	−4.01	−29.87	−4.08	−36.18	−4.23	−36.10	−3.14	−31.29	−5.42	−47.19
Proguanil	−3.73	−22.83	−3.28	−23.24	−3.42	−22.03	−3.39	−24.57	−2.24	−16.06	−4.09	−28.23
Sofosbuvir	−6.12	−63.06	−4.11	−45.41	−4.74	−49.09	−3.48	−43.52	−3.68	−46.41	−6.63	−66.17
Sulfadoxine	−5.13	−36.30	−4.18	−40.51	−2.94	−32.17	−1.00	−23.77	−2.34	−27.77	−3.88	−39.59
Tecovirimat	−3.86	−15.62	−3.51	−35.19	−3.52	−37.36	−3.03	−33.01	−3.05	−26.98	−4.67	−47.96
Tenofovir	−4.23	−42.27	−5.26	−48.79	−4.36	−48.23	−5.07	−66.09	−2.95	−37.36	−5.10	−46.29
Zalcitabine	−5.78	−39.23	−4.92	−31.42	−6.12	−42.38	−3.94	−30.29	−4.27	−34.42	−7.54	−49.09
Zidovudine	−6.35	−45.63	−4.87	−31.49	−5.52	−45.89	−4.12	−36.04	−4.14	−40.12	−5.35	−47.99
MLN4924 (Pevonedistat)	−7.70	−38.62	−2.75	−43.67	−4.62	−52.17	−5.25	−53.68	−4.24	−50.29	−7.21	−71.26
N-acetylcysteine amide	−4.93	−30.64	−4.55	−25.7	−2.04	−28.67	−5.19	−37.26	−3.95	−27.83	−6.55	−42.73
Ritiometan	−2.99	−33.83	−2.29	−27.41	−2.29	−33.50	−2.97	−39.50	−0.57	−16.03	−1.30	−21.01
Rupintrivir	−7.36	−75.11	−1.89	−31.22	−4.36	−55.75	−5.50	−66.86	−4.79	−53.67	−5.62	−68.19
Artemether	−5.14	−19.50	−4.00	−21.98	−3.76	−27.73	−3.39	−23.90	−2.65	−18.13	−5.44	−28.49
Artemisinin	−5.62	−36.63	−4.59	−32.22	−4.13	−33.18	−5.40	−43.61	−3.25	−24.81	−6.28	−43.42
Artenimol	−5.54	−33.43	−4.59	−33.34	−4.38	−35.37	−4.09	−34.63	−3.65	−30.40	−6.27	−49.12
Artesunate	−5.03	−30.97	−4.62	−37.99	−3.51	−33.93	−4.33	−41.08	−3.04	−22.92	−4.66	−38.52
Quinine	−6.26	−44.34	−4.75	−27.38	−4.28	−37.98	−3.73	−38.66	−4.53	−35.73	−5.42	−42.16
Squalene	−3.74	−36.03	−1.80	−28.95	−1.38	−27.35	−0.43	−25.73	−2.78	−34.60	−2.95	−39.73
(−)-Epigallocatechin gallate	−6.19	−56.64	−4.94	−53.06	−5.60	−69.05	−4.50	−53.02	−1.83	−45.05	−5.51	−57.02
24-Ethylcholest-5-en-3beta-ol	−4.64	−33.31	−3.47	−19.58	−3.42	−30.49	−2.18	−22.95	−2.75	−31.31	−5.06	−40.60
Andrograpanin	−4.76	−31.75	−4.08	−24.78	−3.84	−33.38	−3.07	−25.77	−2.59	−25.57	−4.80	−38.85
Andrographiside	−5.42	−48.93	−5.18	−42.37	−4.40	−54.06	−4.50	−43.42	−3.88	−50.19	−5.64	−61.76
Apigetrin (Cosmosiin)	−6.41	−58.72	−5.47	−52.83	−5.15	−56.57	−5.17	−51.21	−3.80	−39.29	−6.21	−64.47
Arctigenin	−5.82	−49.76	−4.60	−36.57	−4.71	−52.73	−2.98	−30.92	−3.12	−37.74	−4.90	−52.28
Baicalin	−6.56	−57.72	−5.79	−64.00	−5.40	−54.77	−5.68	−71.75	−3.70	−50.33	−6.59	−69.05
Berberine	-	-	−4.27	−28.5	−4.26	−38.60	−2.79	−30.34	−3.44	−37.07	−4.89	−42.25
Betulonal	−4.64	−30.53	−3.25	−26.69	−3.24	−39.21	−1.93	−23.81	−2.60	−37.09	−5.22	−40.34
Cerevisterol	−4.90	−28.61	−4.20	−29.31	−3.70	−28.68	−3.45	−33.72	−3.24	−26.82	−5.23	−47.31
Chrysin	−5.78	−39.01	−5.14	−37.68	−4.81	−37.87	−3.51	−29.7	−3.61	−35.94	−5.30	−43.68
Clivimine	-	-	-	-	−3.81	−54.26	−3.58	−48.31	−2.84	−28.49	−3.12	−41.28
Ethyl Ferulate	−5.72	−39.35	−4.09	−26.24	−3.08	−31.06	−2.63	−26.45	−2.60	−26.24	−4.32	−37.72
Ferulic acid	−5.18	−31.36	−4.45	−27.23	−3.69	−28.63	−3.85	−30.05	−2.87	−25.40	−4.31	−32.13
Fucoidan from *Fucus vesiculosus*	−5.01	−33.09	−5.36	−39.24	−4.51	−35.52	−6.20	−58.45	−4.03	−29.66	−5.35	−33.01
Gnidicin	−5.39	−52.85	−2.11	−31.83	−3.39	−47.45	−2.75	−35.15	−2.58	−34.15	−3.66	−48.61
Hesperidin	−6.09	−62.01	−3.67	−45.39	−5.57	−56.62	−5.69	−70.44	−5.26	−71.63	−6.34	−77.63
Honokiol	−5.58	−43.98	−4.23	−33.62	−3.72	−38.67	−2.47	−30.25	−2.77	−30.51	−4.91	−42.44
Kaempferol	−3.13	−43.96	−5.71	−44.83	−4.61	−41.55	−3.89	−35.11	−3.16	−33.24	−6.12	−52.25
Liquiritin	−6.83	−53.66	−5.11	−50.62	−5.14	−55.51	−4.22	−47.62	−4.02	−42.59	−6.42	−66.96
Lycorine	−7.08	−48.82	−5.79	−39.82	−6.12	−46.13	−3.80	−33.97	−4.34	−39.51	−6.41	−50.84
Neohesperidin	−5.84	−55.75	−3.60	−23.29	−5.64	−57.75	−5.42	−68.27	−5.12	−65.39	−6.39	−72.01
Piceatannol	−4.89	−12.54	−5.33	−38.69	−5.04	−36.70	−4.16	−34.80	−4.04	−32.36	−5.48	−49.47
Rosmarinic acid	−5.53	−52.06	−5.44	−52.15	−4.95	−59.89	−4.87	−54.99	−3.88	−39.13	−5.05	−56.03
Theaflavine-3,3′-digallate-3,3′-digallate	-	-	-	-	−5.57	−68.47	−6.43	−79.69	−5.53	−56.23	−6.50	−103.39
Ursolic acid	−3.46	−35.24	−3.78	−31.9	−2.93	−32.34	0.01	−31.42	−2.31	−32.93	−4.54	−38.30
α-Mangostin	−4.29	−50.96	−1.03	−37.36	−3.48	−41.76	−0.63	−38.95	−3.57	−45.4	−4.69	−52.43

**Table 7:** Compounds consistently identified among the top 20 docking candidates across the five ISKNV target proteins were selected for subsequent *in vitro* evaluation.

Compound Name	DNA Polymerase		TFIIS		ATPase 1		ATPase 2		Ankyrin Repeat-Containing Protein		MCP		Selected for *In Vitro* Evaluation
	Docking	E-Model	Docking	E-Model	Docking	E-Model	Docking	E-Model	Docking	E-Model	Docking	E-Model	
Fludarabine	−6.48	−46.42	−5.11	−39.17	−5.48	−49.01	−5.68	−52.06	−4.57	−34.15	−6.78	−52.95	Yes
Liquiritin	−6.83	−53.66	−5.11	−50.62	−5.14	−55.51	−4.22	−47.62	−4.02	−42.59	−6.42	−66.96	Yes
Lycorine	−7.08	−48.82	−5.79	−39.82	−6.12	−46.13	−3.80	−33.97	−4.34	−39.51	−6.41	−50.84	Yes

**Table 8:** Summary of the principal ligand–protein interactions identified from representative molecular docking poses of selected antiviral compounds with ISKNV target proteins. Hydrogen bonding and hydrophobic interactions were identified from two-dimensional interaction maps generated using the Schrödinger Maestro Glide module.

Target Protein	Compound	Hydrogen Bond Residues	Hydrophobic Interaction Residues
DNA polymerase	Fludarabine	Tyr221, Tyr274	Ile207, Ile223, Val216, Val220, Val365, Leu277, Leu421, Tyr221, Tyr274
	Liquiritin	Tyr221, Lys219	Tyr221, Tyr274, Ile207, Ile223, Val216, Val220, Val222, Val365, Cys211, Leu421
	Lycorine	Tyr221, Lys219	Tyr221, Tyr274, Val216, Val220, Val365, Ile207, Ile223, Cys211, Leu421
TFIIS	Fludarabine	Glu15, Gln18, Ala66	Ala16, Ala22, Ala66, Val65, Leu21
	Liquiritin	Glu15, Asp19, Lys43	Ala22, Ala66, Val65, Leu21, Tyr12
	Lycorine	Gln18, Ala66, Val65	Ala22, Ala66, Val65, Phe26
ATPase 1	Fludarabine	Arg12, Thr204, Arg207, Asp212, Arg215	Leu11, Tyr200, Met20, Met23, Met213, Leu6
	Liquiritin	Glu10, Asp18, Asp202, Asp212, Arg215	Met20, Met213, Val14, Leu6, Leu11, Tyr200
	Lycorine	Glu10, Asp202, Arg207	Met20, Met23, Leu6, Leu11, Val14, Tyr200
ATPase 2	Fludarabine	Asp110, Asp146	Met112, Tyr143
	Liquiritin	Asp110, Asp113, Lys171, Gln142	Met112, Tyr143, Pro31, Cys168
	Lycorine	Asp110, Asp113	Met112, Tyr143
Ankyrin repeat-containing protein	Fludarabine	Leu195, Asp190, Arg221	Leu195, Pro194, Phe217
	Liquiritin	Asp190, Arg221	Phe187, Val204, Ala205, Phe217, Leu195, Pro194
	Lycorine	Glu197, Arg221	Pro194, Leu195, Phe217
MCP	Fludarabine	Glu369, Asn133, Asn146, Asp130	Met151, Met370, Leu129, Leu326, Val373, Tyr312, Ala134
	Liquiritin	Leu326, Asn103, Asn133, Asn146	Met151, Met370, Ala134, Ile149, Leu326, Val324, Val373, Tyr312
	Lycorine	Glu369, Asn103, Asn133	Met151, Met370, Ala134, Val373, Phe131, Tyr312, Leu326

**Table 9:** Inhibition rates of selected compounds against MCP, ATPase, and DNA polymerase gene expression compared to the ISKNV-infected control group at 72 h post-infection.

Compound Name	Concentrations (μg/mL)	MCP Inhibition Rate (%) (Mean ± SD)	ATPase Inhibition Rate (%) (Mean ± SD)	DNA Polymerase Inhibition Rate (%) (Mean ± SD)
Fludarabine	100	91.67 ± 0.72	90.66 ± 0.35	86.11 ± 0.82
Liquiritin	100	52.11 ± 5.60	50.33 ± 5.16	56.41 ± 2.59
Lycorine	0.5	99.81 ± 0.08	99.66 ±0.09	85.46 ± 4.33

## Data Availability

The data presented in this study are available upon request from the corresponding author.

## Abbreviations

Abbreviations used in this manuscript are as follows:

ISKNV	Infectious spleen and kidney necrosis virus
RSIV	Red sea bream iridovirus
ORFs	Open reading frames
DGF	Dwarf gourami fin
CPE	Cytopathic effect
CID	Compound identifier
CAS	Chemical Abstracts Service
TFIIS	Transcription elongation factor
ATPase	Adenosine triphosphatase
MCP	Major capsid protein
PCR	Polymerase chain reaction
PBD	Protein Data Bank
SP	Standard precision
L-15	Leibovitz’s
FBS	Fetal bovine serum
NEAA	Non-essential amino acids
HEPES	Hydroxyethyl piperazine ethane sulfonic acid
MCE	MedChemExpress
DMSO	Dimethyl sulfoxide
TCID_50_	Median tissue culture infectious dose
PBS	Phosphate-buffered saline
ANOVA	Analysis of variance

## Appendix A

**Table A1:** Amino acid sequences of selected ISKNV proteins used for molecular docking analysis. aa: Amino Acid; *: stop codon.

Protein Name	ORF No. (AF371960)	Amino Acid Length	Amino Acid Sequence
DNA polymerase	ORF019R	948 aa	MDSVYIYQWLYANYEVRGYGIAPNNTVVCVRVPNFKQVVYVECTDPQQHDPRSTFTQHGFRVYETPRACSLYGAKGVGTYFAARVPNYNAMRDVQETQGPFKIHESRVSKTMEFTARAGLPTVGWIQVSQRCVVTRTVTMAAKEYMVPNWRTDVRPAPDMEGVPPAKIVYFDIEVKSDHENVFPSDRDDEVIFQIGLVLCSGNTVLRTDLLSLPGRDYDDSVYQYATEGELLHAFIAYIREHEVVAVCGYNIMGFDIPYIIKRCARTSMLGTLRRIGFDNRRLAIEKTAGVGHAKMTYIQWEGVLTIDLMPIIMMDHKLDSYSLDYVANHFVKAGKDPIRPRDIFHAYNTGMMAPVGAYWLKEPQLCKQLVDYLNTWVALCEMAGVCNTSIMQLFTQGQQVRVFAQIYRDCTPMDVVDKVYVIPDGGCDSDVVSPSSYTGAYVYEPVPGVYKNVIPMDFQSLYPSIIISKNICYSTLVDQGGEEYAWQEHEGCEHDPQYAKQHALGIEIGVLQCNMAALPRRATQERARLRERIADMKIQYASMTPAAVKCNVFSFRFTHAHEGVLPRVLRNLLESRARIRARIKTTDDPDIRAVLDKRQLAYKISANSVYGTMGTQRGYLPFMAGAMTTTYCGRKLIEKAAHLLKTVVGATIVYGDTDSCYIQLGHDRASLDELWQMAVNASDTVSAFFERPVRLEFEQCIYTKFIIFTKKRYVYRAFTRDGKQRTGSKGVMLSRRDSAMCARNTYAAIMNTILEGSADVPFIAACMMHDMMIPGALQDDDFVLTKSVQDIGNGDDNNQGSYKVRNPQKAQAAATQRVAPDDAEGYAIALRQEMVKQMPAQAQLAERMRLQGRAVVSGARIEYVVLKHQYGVPEGALGARLLDFERWREMKVAYPLDRLYYMKSVVNACDQLLVTAGYGPVCSKVYAAHLQLAYVHKQLLRRTTPAV *
TFIIS	ORF029L	73 aa	MYTCTTMNKDLYDKEAEQDRLARTRFSSLTQSQYLCRACGNAKTYTLTMQTRGGDEALSVFVCCVACGKRYRI*
ATPase	ORF122R	239 aa	MEIKELSLTELRPVKPDDEMGGMKLIVLGKPQRGKSVLIKSIIAAKRHIIPAAVVISGSEEANHFYSKLLPNCFVYNKFDADIITRVKQRQLALKNVDPEHSWLMLIFDDCMDNAKMFNHEAVMDLFKNGRHWNVLVIIASQYIMDLNASLRCCIDGVFLFTETSQTCVDKIYKQFGGNIPKQTFHTLMEKVTQDHTCLYIDNTTTRQKWEDMVRYYKAPLLTDADVGFGFKDYKAGVA*
Ankyrin repeat-containing protein	ORF125L	228 aa	MLPEELAGALRTGGSNGQSILFDAIRSGTITAFTGVHADIVNTVREHSTGNTLLMAAVMTRDLLIIKHVVENLGYNTFMGMRLSDHATALHLVACLADPYHGCVRYLLTQYGAQMAPALTMTTNEDDMNPLHYACKYGGVQTMVLLATVMSQYDGFAQACFALNASLAQPALLALHYDELGGAQKAFILDSVAPLQERWNGHNVARLIRGDHNLTWFLMARNNKDFFA*
MCP	ORF006L	453 aa	MSAISGANVTSGFIDISAFDAMETHLYGGDNAVTYFARETVRSSWYSKLPVTLSKQTGHANFGQEFSVTVARGGDYLINVWLRVKIPSITSSKENSYIRWCDNLMHNLVEEVSVSFNDLVAQTLTSEFLDFWNACMMPGSKQSGYNKMIGMRSDLVAGITNGQTMPAVYLNLPIPLFFTRDTGLALPTVSLPYNEVRIHFKLRRWEDLLISQSNQADMAISTVTLANIGNVAPALTNVSVMGTYAVLTSEEREVVAQSSRSMLIEQCQVAPRVPVTPADNSLVHLDLRFSHPVKALFFAVKNVTHRNVQSNYTAASPVYVNNKVNLPLMATNPLSEVSLIYENTPRLHQMGVDYFTSVDPYYFAPSMPEMDGVMTYCYTLDMGNINPMGSTNYGRLSNVTLSCKVSDNAKTTAAGGGDNGSGYTVAQKFELVVIAVNHNIMKIADGAAGFPIL*
